# Impact of red blood cell distribution width on response to HIF-PH inhibitor in improving anemia and clinical outcomes in patients with chronic kidney disease

**DOI:** 10.1186/s12882-025-04675-2

**Published:** 2025-12-06

**Authors:** Sayaka Murai, Kota Kakeshita, Teruhiko Imamura, Tsutomu Koike, Hidenori Yamazaki, Koichiro Kinugawa

**Affiliations:** The Second Department of Internal Medicine, University of Toyama, 2630 Sugitani, Toyama, Toyama, 930-0194 Japan

**Keywords:** Renal anemia, RDW-CV, Mean corpuscular volume, Iron deficiency, ESA resistance, Heart failure

## Abstract

**Background:**

The clinical feasibility and efficacy of hypoxia-inducible factor prolyl hydroxylase (HIF-PH) inhibitors in patients with anemia in chronic kidney disease (CKD) remain uncertain. Red blood cell distribution width (RDW) may serve as a potential predictor for responsiveness to HIF-PH inhibitor therapy.

**Methods:**

Consecutive patients receiving HIF-PH inhibitors for anemia in CKD over three months were included. The predictive value of baseline RDW for the improvement in anemia in CKD, defined by the increase in hemoglobin levels to 10.0 g/dL or higher at within the first three months after initiation of therapy and the maintenance of that level thereafter, was assessed.

**Results:**

A total of 106 patients were enrolled (median age 79 years; 57 males; 75 patients with heart failure; median estimated glomerular filtration rate 25.0 mL/min/1.73 m²; median hemoglobin level 9.3 g/dL). Among these, 84 patients met the primary outcome criteria. Baseline RDW-coefficient of variation (CV) ranged from 11.8% to 22.4%, with a median of 14.0%. Baseline RDW-CV independently predicted the primary outcome, with an odds ratio of 2.43 (95% confidence interval: 1.42–4.14, *p* < 0.01). The optimal cutoff value for predicting the primary outcome was identified as 13.7% (sensitivity 0.67, specificity 0.86). In addition, baseline RDW-CV levels were not significantly associated with overall and renal-related prognosis.

**Conclusions:**

Baseline RDW-CV independently predicts the therapeutic efficacy of mid-term HIF-PH inhibitor treatment for anemia in CKD.

**Trial registration:**

Not applicable.

**Supplementary Information:**

The online version contains supplementary material available at 10.1186/s12882-025-04675-2.

## Introduction

Anemia is a prevalent complication among patients with chronic kidney disease (CKD). Anemia in CKD arises from multifactorial etiologies, including reduced endogenous erythropoietin synthesis, iron deficiency, and inflammation-induced hepcidin [1]. The prevalence of anemia in CKD increases concomitantly with CKD progression [2]. Anemia in CKD is significantly associated with increased morbidity and mortality, including cognitive dysfunction, insomnia, cardiovascular events, hospital readmissions, deterioration in quality of life, and further CKD progression [3].

Erythropoiesis-stimulating agents (ESAs) have traditionally served as primary therapeutic agents for renal anemia. Generally, clinical outcomes of ESA therapy have been satisfactory in patients with pre-dialysis CKD or those dependent on dialysis, whereas some patients were refractory to ESAs [4], probably due to chronic inflammation [5]. Under the condition of chronic inflammation, increased hepcidin induces functional iron deficiency [6]. Conversely, the optimal control of hemoglobin levels during ESA therapy is sometimes challenging. An extremely elevated hemoglobin levels during ESA therapy rather increase the risk of cardiovascular death [7].

Since 2020, hypoxia-inducible factor prolyl hydroxylase (HIF-PH) inhibitors have emerged as novel therapeutic options for patients with renal anemia [8–12]. HIF-PH inhibitors are orally administered, instead of ESAs. HIF-PH inhibitors function by activating HIF and stimulating endogenous erythropoietin production [13]. HIF-PH inhibitors improve functional iron deficiency by facilitating the section of endogenous erythropoietin and suppressing the synthesis of hepcidin [14]. As a result, HIF-PH inhibitors have the potential to treat anemia in CKD refractory to conventional ESAs.

Nonetheless, variability exists in responsiveness to HIF-PH inhibitor therapy. HIF-PH inhibitors excessively increase hemoglobin levels in some patients, whereas others are refractory to HIF-PH inhibitors. These are probably due to heterogeneous patients’ backgrounds. The identification of potential predictions for the responsiveness to HIF-PH inhibitors should be helpful for optimal patient selection.

Red blood cell distribution width (RDW) is a hematological parameter that quantifies the variation in erythrocyte volume. RDW serves as an effective diagnostic tool for elucidating anemia etiologies. Elevated RDW levels reflect nutritional deficits, iron deficiency [15], systemic inflammation [16, 17], or ESA resistance [18], being associated with increased cardiovascular morbidity [19].

Experimental studies in renal anemia models have demonstrated the concomitant increases in hemoglobin and RDW levels following HIF-PH inhibitor administration [20, 21]. RDW represents the status of iron deficiency and chronic inflammation, and may theoretically be considered as a potential predictor of responsiveness to HIF-PH inhibitors. However, clinical investigations elucidating the relationship between RDW and HIF-PH inhibitors are absent. Therefore, this study aims to investigate the influence of baseline RDW levels on therapeutic response to HIF-PH inhibitors in patients with CKD-associated anemia.

## Methods

### Patient selection

Patients diagnosed with CKD who commenced HIF-PH inhibitor therapy for anemia for the first time and maintained treatment for a minimum of three months between December 2020 and March 2025 were eligible for inclusion. Exclusion criteria comprised patients with apparent active bleeding or those requiring regular dialysis therapy. The study protocol was reviewed and approved by the Institutional Ethics Committee (approval number R2024206, dated February 14, 2025). Given the retrospective design and the adoption of an opt-out approach, the requirement for informed consent was waived. The study adhered strictly to the principles outlined in the Declaration of Helsinki.

### Study protocol

Data on the included patients were followed following the initiation of HIF-PH inhibitors (day 0) during the therapeutic period until the termination of HIF-PH inhibitors or the end of study period (July 2025).

The dosages of HIF-PH inhibitors were adjusted to achieve the maintenance range of hemoglobin between 10.0 g/dL and 13.0 g/dL, according to the manufacturer-recommended regimens. Iron was orally supplied appropriately in case of iron deficiency with serum ferritin less than 100 ng/mL and/or transferrin saturation less than 20%. The types of HIF-PH inhibitors were selected from agents administrated once daily; Daprodustat (Kyowa Kirin Co., Ltd., Tokyo, Japan), Vadadustat (Mitsubishi Tanabe Pharma Corporation, Osaka, Japan), Enarodustat (Torii Pharmaceutical Co., Ltd., Tokyo, Japan), and Molidustat (Bayer Yakuhin, Ltd., Osaka, Japan).

The independent variable evaluated was the baseline RDW-coefficient of variation (RDW-CV). The primary endpoint of this study was the improvement in hemoglobin levels, specifically defined as an increase in hemoglobin levels of 10.0 g/dL or higher within the first three months after initiation of therapy and being maintained thereafter. Secondary endpoints included an excessive increase in hemoglobin levels ≥ 13.0 g/dL, all-cause mortality or progression to end-stage renal disease (only the first one was counted as an achievement of endpoint), ≥ 50% decline in estimated glomerular filtration rate (GFR), and ≥ 25% decline in estimated GFR. The estimated GFRs were measured successively and the date when the endpoint was achieved for the first time was counted as an achievement of endpoint.

### Data collection

Baseline characteristics obtained just before the initiation of HIF-PH inhibitors were retrieved, including demographics, comorbidity, laboratory, urine, medication, and vital signs data. The etiology of CKD was determined by the attending nephrologists. The diagnosis of diabetes mellitus was defined as a history of any therapeutic interventions.

### Calculation of laboratory parameters

Baseline RDW values just before the initiation of HIF-PH inhibitors were retrieved as independent variables. RDW-standard deviation (RDW-SD) was derived from histogram plots, where cell count is displayed on the y-axis and red blood cell volume (fL) on the x-axis. RDW-SD reflects the degree of anisocytosis and is defined as the width of the red blood cell volume distribution curve at the 20% height level. Unlike RDW-CV, RDW-SD is not influenced by the mean corpuscular volume (MCV); however, it is less suitable for comparing anemias with markedly different MCV values. We used RDW-CV, instead of RDW-SD, because we assessed individuals with a variety of MCVs.

RDW-CV expresses the relative variation in red blood cell size as a percentage, representing the extent of size heterogeneity relative to the MCV. The theoretical RDW-CV (%) is calculated using the formula: (SD of red blood cell volume [fL] / MCV [fL]) × 100. The reference range for RDW-CV in healthy individuals is 11.5–14.5% [22].

In practice, red blood cell distributions are often skewed, as in iron deficiency anemia. In this study, we adopted the measurement method of the XN-9100 analyzer (Sysmex Corporation, Kobe, Japan). The RDW-CV measured by this analyzer is calculated by determining two values, length 1 (L1) and length 2 (L2), positioned symmetrically around the histogram peak so that 68% of the distribution is included (assuming SD). RDW-CV is then calculated as (L2 − L1) / (L2 + L1) × 100, thereby approximating the theoretical RDW-CV. This method allows measurement that more closely reflects the actual red blood cell distribution and is therefore clinically useful.

The estimated GFR was calculated as follows: 194 × (serum creatinine [mg/dL]) ^−1.094^ × (age [years]) ^−0.287^ (× 0.739 only for women) [23].

### Statistical analyses

All statistics were performed using EZR (Saitama Medical Center, Jichi Medical University, Saitama, Japan). *P* value < 0.050 was assumed as a statistical significance. Given the moderate sample size, continuous variables were dealt as non-parametric variables irrespective of their distribution pattern. They were expressed as median (25th percentile, 75th percentile) and compared between the two groups using Mann-Whitney *U* test. Categorical variables were expressed as numbers (percentages) and compared between the two groups using Fischer’s exact test. Plasma B-type natriuretic peptide (BNP) and serum C-reactive protein (CRP) values were log₁₀-transformed before analysis because of their skewed distributions.

The independent variable was defined as baseline RDW-CV. The primary outcome was the achievement of increasing and maintained hemoglobin level ≥ 10.0 g/dL. The prognostic impact of the independent variable was evaluated by the logistic regression analysis, by adjusting for potential confounders, including age, sex, estimated GFR, serum albumin, hemoglobin, transferrin saturation, logarithm of serum CRP, logarithm of plasma BNP, and the type of HIF-PH inhibitors, according to their association with RDW-CV and prognostic impacts.

Receiver operating characteristics analysis was performed to calculate a cutoff of RDW-CV for the primary outcome. The whole cohort was divided into two groups based on the calculated cutoff. The trajectory of hemoglobin levels and RDW-CV was assessed by using Friedman test in the two groups.

The impact of RDW-CV on the excessive increase of hemoglobin level ≥ 13.0 g/dL was evaluated by Cox proportional hazard ratio regression analyses. A cutoff of RDW-CV for this secondary outcome was calculated in the same manner with the primary outcome. The cumulative incidences of this outcome were compared between the two groups by the log-rank test.

The prognostic impact of RDW-CV on other secondary outcomes (all-cause death or end-stage renal disease, ≥ 50% decline of estimated GFR, and ≥ 25% decline of estimated GFR) was evaluated by Cox proportional hazard ratio regression analyses.

## Results

### Baseline characteristics

A total of 106 patients were included (Table 1). Median age was 79 [72–85] years and 57 were men. Median estimated GFR was 25.0 [17.0–33.3] mL/min/1.73 m^2^ and median hemoglobin level was 9.3 [8.8–9.8] g/dL. Seventy-five (71%) patients had heart failure.

**Table 1:** Baseline characteristics

Variables	Total (*N* = 106)	Low RDW-CV (< 13.7%) (*n* = 47)	High RDW-CV (≥ 13.7%) (*n* = 59)	*p* value
**Demographics**				
Age, years	79 [72–85]	78 [69–85]	79 [75–86]	0.23
Male sex	57 (54%)	26 (55%)	31 (53%)	0.85
Current smoker	4 (4%)	2 (4%)	2 (3%)	1.00
Past smoker	39 (37%)	16 (34%)	23 (39%)	0.54
Body mass index, kg/m^2^	21.5 [19.1–23.9]	22.3 [19.5–25.9]	21.3 [18.8–23.5]	0.13
Systolic blood pressure, mmHg	118 [104–134]	120 [105–132]	117 [104–134]	0.66
Diastolic blood pressure, mmHg	66 [57–76]	64 [53–77]	66 [57–75]	0.80
Pulse rate, bpm	73 [65–82]	74 [66–83]	73 [66–82]	0.85
**Comorbidity**				
Chronic heart failure	75 (71%)	27 (57%)	48 (81%)	< 0.01*
Coronary artery disease	34 (32%)	13 (28%)	21 (36%)	0.41
Atrial fibrillation	39 (37%)	11 (23%)	28 (48%)	0.015*
Diabetes mellitus	32 (30%)	12 (26%)	20 (34%)	0.40
History of stroke	14 (13%)	9 (19%)	5 (9%)	0.15
**CKD stage**				< 0.01*
Stage G3, estimated GFR 30–59 mL/min/1.73 m^2^	38 (36%)	11 (23%)	27 (46%)	-
Stage G4, estimated GFR 15–29 mL/min/1.73 m^2^	47 (44%)	21 (45%)	26 (44%)	-
Stage G5, estimated GFR < 15 mL/min/1.73 m^2^	21 (20%)	15 (32%)	6 (10%)	
**The primary diseases of CKD**				0.17
Chronic glomerulonephritis	13 (12%)	8 (17%)	5 (9%)	-
Nephrosclerosis	56 (53%)	21 (45%)	35 (59%)	-
Diabetic nephropathy	20 (19%)	7 (15%)	13 (22%)	-
Polycystic kidney disease	3 (3%)	2 (4%)	1 (3%)	-
Other or unknown	14 (13%)	9 (19%)	5 (9%)	-
**Laboratory data**				
Red blood cells, ×10^6^/µL	303 [284–325]	304 [294–320]	300 [278–326]	0.35
Hemoglobin, g/dL	9.3 [8.8–9.8]	9.5 [9.2–9.9]	8.9 [8.6–9.7]	< 0.01*
Hematocrit, %	28.7 [27.4–30.4]	29.0 [28.1–31.5]	28.2 [26.8–30.1]	0.022*
Mean corpuscular volume, fL	95.8 [91.2–99.0]	96.4 [92.9–98.7]	94.3 [89.7–99.8]	0.14
Mean corpuscular hemoglobin, pg	30.7 [29.6–32.1]	31.0 [30.2–32.1]	30.4 [28.5–31.7]	0.044*
Mean corpuscular hemoglobin concentration, %	32.0 [31.4–32.8]	32.2 [31.7–33.2]	32.0 [31.4–32.7]	0.16
RDW-SD, fL	48.3 [44.9–52.0]	45.1 [42.9–46.8]	50.8 [49.0–54.2]	< 0.01*
RDW-CV, %	14.0 [13.0–14.8]	12.9 [12.5–13.2]	14.7 [14.2–15.8]	< 0.01*
Serum albumin, g/dL	3.6 [3.2–3.9]	3.8 [3.5–4.0]	3.5 [2.9–3.8]	< 0.01*
Estimated GFR, mL/min/1.73 m^2^	25.0 [17.0–33.3]	20.1 [13.5–29.6]	28.0 [20.6–36.0]	< 0.01*
Serum ferritin, ng/mL	109 [63–218]	134 [81–216]	88 [42–215]	0.24
Transferrin saturation, %	24.4 [15.5–30.4]	26.8 [21.8–33.6]	19.9 [12.1–27.6]	< 0.01*
Serum C-reactive protein, mg/dL	0.22 [0.04–0.48]	0.08 [0.03–0.27]	0.28 [0.12–0.66]	< 0.01*
Plasma B-type natriuretic peptide, pg/mL	186.6 [94.7–347]	131.0 [68.9–246.7]	210.1 [115.4–441.5]	0.039*
Urine protein, g/g of creatinine	0.62 [0.19–2.09]	0.83 [0.28–2.37]	0.41 [0.16–1.50]	0.30
**Medications**				
ACE inhibitor or ARB	61 (58%)	29 (62%)	32 (54%)	0.55
Angiotensin receptor-neprilysin inhibitor	31 (29%)	11 (23%)	20 (34%)	0.29
Mineralocorticoid receptor antagonist	36 (34%)	9 (19%)	27 (46%)	< 0.01*
Sodium-glucose cotransporter 2 inhibitor	28 (26%)	11 (23%)	17 (29%)	0.66
Beta-adrenergic blocker	69 (65%)	30 (64%)	39 (66%)	0.84
Switching from ESAs	25 (24%)	11 (23%)	14 (24%)	1.00
Oral iron supplements	36 (34%)	10 (21%)	26 (44%)	0.023*
**HIF-PH inhibitors**				0.045*
Daprodustat	67 (63%)	24 (51%)	43 (73%)	-
Vadadustat	31 (29%)	17 (36%)	14 (24%)	-
Enarodustat	5 (5%)	3 (6%)	2 (3%)	-
Molidustat	3 (3%)	3 (6%)	0 (0%)	-

RDW indicates red cell distribution width; CV, coefficient of variation; CKD, chronic kidney disease; GFR, glomerular filtration rate; SD, standard deviation; ACE, angiotensin converting enzyme; ARB, angiotensin II receptor blocker; ESA, erythropoiesis stimulating agent; HIF-PH, hypoxia inducible factor-prolyl hydroxylase

Baseline characteristics data were obtained at the time when HIF-PH inhibitors were initiated. Continuous variables are presented as median [25th percentile–75th percentile] and compared between the two groups using Mann-Whitney *U* test. Categorical variables are presented as numbers (percentage) and compared between the two groups using Fisher’s exact test. *p < 0.05

Twenty-five (24%) patients had conversion from ESAs to HIF-PH inhibitors. The hemoglobin levels at the conversion ranged between 8.1 and 13.3 g/dL. The types of HIF-PH inhibitors consisted of 67 Daprodustat, 31 Vadadustat, 5 Enarodustat, and 3 Molidustat.

Patients had a variety of etiologies for CKD, including 13 chronic glomerulonephritis, 56 nephrosclerosis, 20 diabetic nephropathy, 3 polycystic kidney disease, and 14 other or unknown origins.

### Baseline RDW-CV

RDW-CVs were distributed between 11.8% and 22.4% with a median value of 14.0 [13.0–14.8] % (Supplementary Fig. 1). A higher RDW-CV was correlated with more progressed anemia indicated by lower hemoglobin (*r* = − 0.29, *p* < 0.01; Supplementary Fig. 2a). RDW-CV was not significantly correlated with estimated GFR (*p* = 0.08; Supplementary Fig. 2b).

### Impact of baseline RDW-CV on change in hemoglobin

**Fig. 1:**
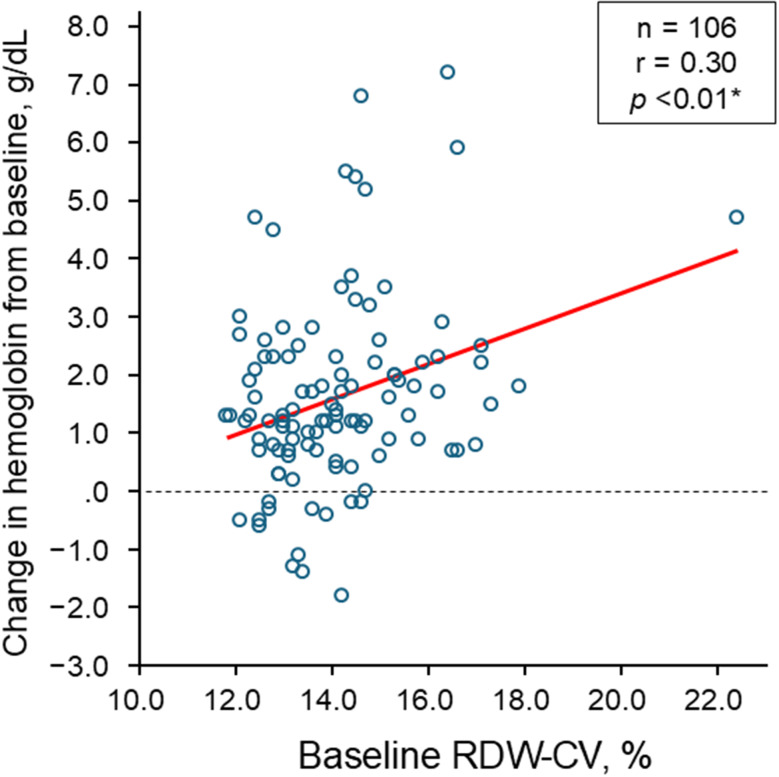
Correlation between baseline RDW-CV and change in hemoglobin during the first 3-month therapeutic period. RDW indicates red cell distribution width; CV, coefficient of variation *p < 0.05 by Pearson’s correlation coefficient

Baseline RDW-CV levels showed a positive correlation with the change in hemoglobin levels from baseline during the 3-month HIF-PH inhibitors therapy (*r* = 0.30, *p* < 0.01; Fig. 1). A similar trend of correlation was observed in patients with conversion from ESA (*r* = 0.33, *p* = 0.11; Supplementary Fig. 3).

### Impact of baseline RDW-CV on the primary outcome

**Fig. 2:**
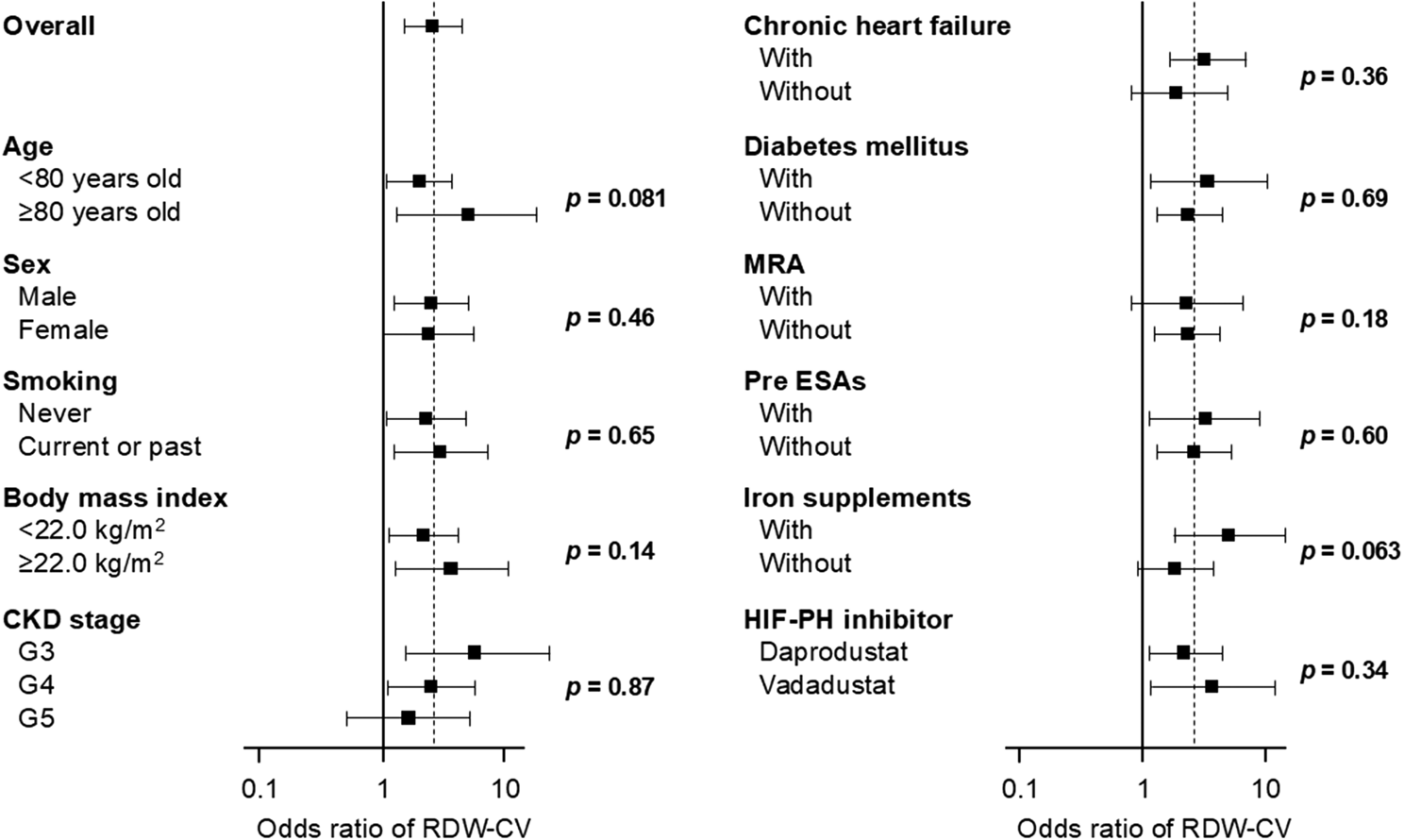
Impact of RDW-CV to predict the primary concern in subgroups. RDW indicates red cell distribution width; CV, coefficient of variation; CKD, chronic kidney disease; MRA, mineralocorticoid receptor antagonist; ESA, erythropoiesis-stimulating agent; HIF-PH, hypoxia-inducible factor-prolyl hydroxylase. Black boxes represent odds ratios and bars represent a 95% confidence interval

During the first 3-month HIF-PH inhibitor therapy period, 84 patients achieved the primary endpoint: increasing and maintaining hemoglobin levels of 10.0 g/dL or higher. Baseline RDW-CV was a significant predictor on the primary outcome with a crude odds ratio of 2.43 (95% confidence interval 1.42–4.14, *p* < 0.01; Table 2). RDW-CV remained an independent predictor for achieving the primary outcome after adjusting for potential confounders, including age, sex, estimated GFR, serum albumin, hemoglobin, transferrin saturation, logarithm of serum CRP, logarithm of plasma BNP, and the type of HIF-PH inhibitors (*p* < 0.01 for each model; Table 2). A similar trend was consistently observed across most subgroups (interaction *p* > 0.05 for all sub-groups; Fig. 2).

**Table 2:** Impact of baseline RDW-CV to predict the improvement of anemia

Explanatory variables	Odds ratio	95% confidence interval	*p* value
**Univariable analysis**			
Age, years	1.02	0.99–1.05	0.33
Male sex	0.96	0.37–2.47	0.94
Body mass index, kg/m^2^	1.02	0.91–1.15	0.69
Estimated GFR, mL/min/1.73 m^2^	0.99	0.95–1.03	0.67
Serum albumin, g/dL	0.32	0.12–0.89	0.030*
Hemoglobin, g/dL	0.41	0.24–0.70	< 0.01*
Serum ferritin, ng/mL	1.00	1.00–1.00	0.96
Transferrin saturation, %	0.94	0.89–1.00	0.036*
Logarithm of serum CRP, mg/dL	2.26	1.08–4.74	0.030*
Logarithm of plasma BNP, pg/mL	1.32	0.40–4.35	0.65
Mean corpuscular volume, fL	0.98	0.91–1.06	0.64
RDW-CV, %	2.43	1.42–4.14	< 0.01*
Switching from ESAs	0.44	0.16–1.23	0.12
Oral iron supplements during the first 3 months	0.83	0.32–2.11	0.69
**Multivariable analysis**			
**Model 1 (Age + Sex)**			
RDW-CV, %	2.48	1.42–4.32	< 0.01*
**Model 2 (Model 1 + Estimated GFR)**			
RDW-CV, %	3.02	1.60–5.68	< 0.01*
**Model 3 (Model 1 + Serum albumin)**			
RDW-CV, %	2.23	1.27–3.92	< 0.01*
**Model 4 (Model 1 + Hemoglobin)**			
RDW-CV, %	2.44	1.32–4.52	< 0.01*
**Model 5 (Model 1 + Transferrin saturation)**			
RDW-CV, %	2.60	1.35–5.02	< 0.01*
**Model 6 (Model 1 + Logarithm of serum CRP)**			
RDW-CV, %	2.20	1.24–3.92	< 0.01*
**Model 7 (Model 1 + Logarithm of plasma BNP)**			
RDW-CV, %	3.00	1.50–5.97	< 0.01*
**Model 8 (Model 1 + The type of HIF-PH inhibitors)**			
RDW-CV, %	2.44	1.38–4.31	< 0.01*

RDW indicates red cell distribution width; CV, coefficient of variation; GFR, glomerular filtration rate; CRP, C-reactive protein; BNP, B-type natriuretic peptide

*p < 0.05 by logistic regression analyses

### Cutoff of RDW-CV for the primary outcome

**Fig. 3:**
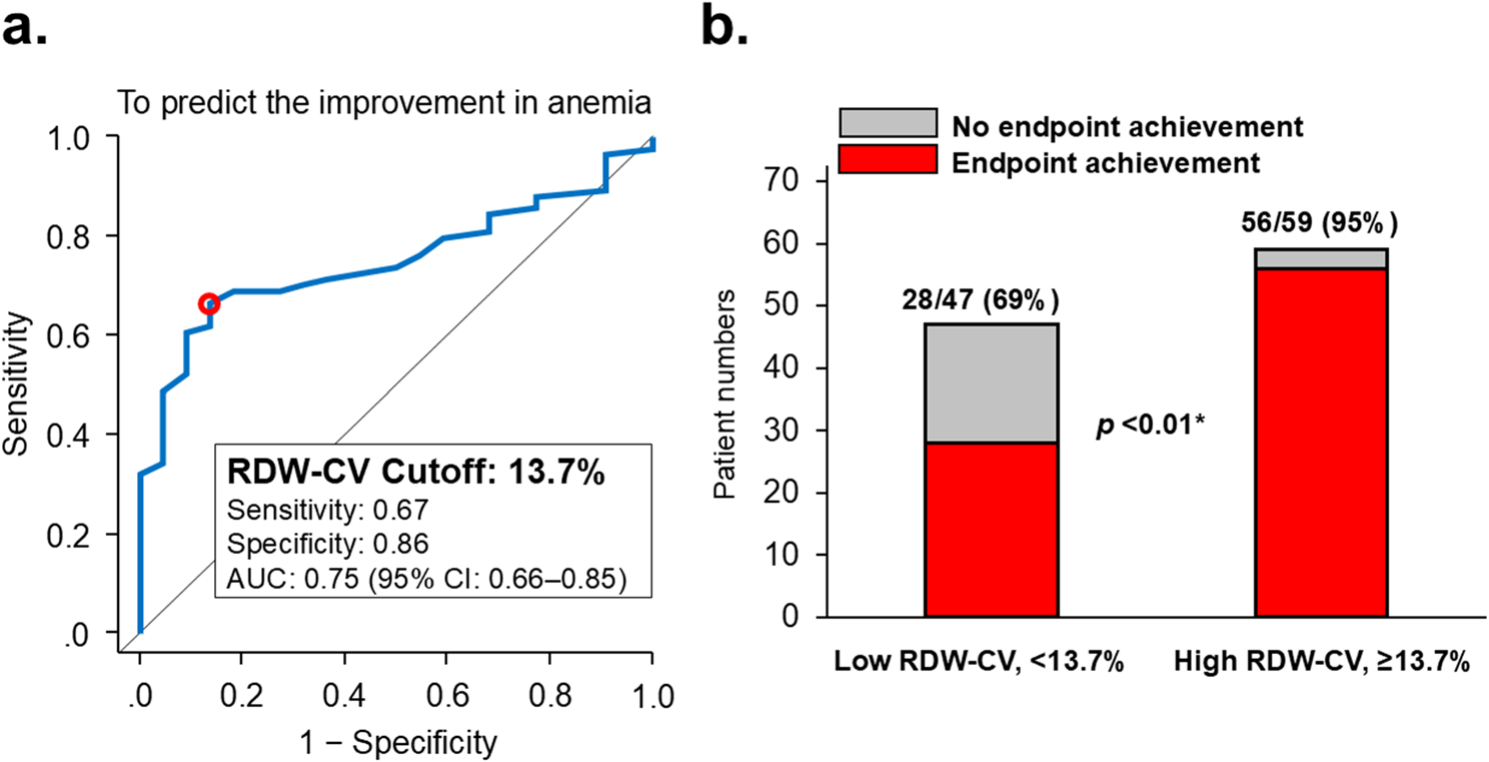
Receiver operating characteristics analysis (**a**); comparison of patient numbers achieving the primary endpoint (**b**). (**a**) Receiver operating characteristics analysis to calculate a cutoff value of RDW-CV for the primary concern. (**b**) The numbers of patients who achieved the primary endpoint stratified by the calculated cutoff. RDW indicates red cell distribution width; CV, coefficient of variation; AUC, area under the curve; CI, confidence interval. *p < 0.05 by Chi-square test

The optimal cutoff value of RDW-CV for predicting the primary outcome was determined to be 13.7%, yielding an area under the receiver operating characteristic curve of 0.75 (95% confidence interval 0.66–0.85), sensitivity of 0.67, and specificity of 0.86 (Fig. 3a). The predictive performance of RDW-CV surpassed other parameters such as MCV (Supplementary Fig. 4). As a result, 56/59 (95%) in the high RDW-CV group achieved the primary endpoint, whereas 28/47 (69%) patients in the low RDW-CV group achieved the primary endpoint (*p* < 0.10; Fig. 3b).

Patients were stratified into two groups based on the RDW-CV cutoff. Patients with higher RDW-CV exhibited a significantly greater prevalence of heart failure and lower prevalence of advanced-stage CKD compared to those with lower RDW-CV (*p* < 0.05 for all; Table 1).

### Trajectory of hemoglobin level changes stratified by the cutoff of RDW-CV

**Fig. 4:**
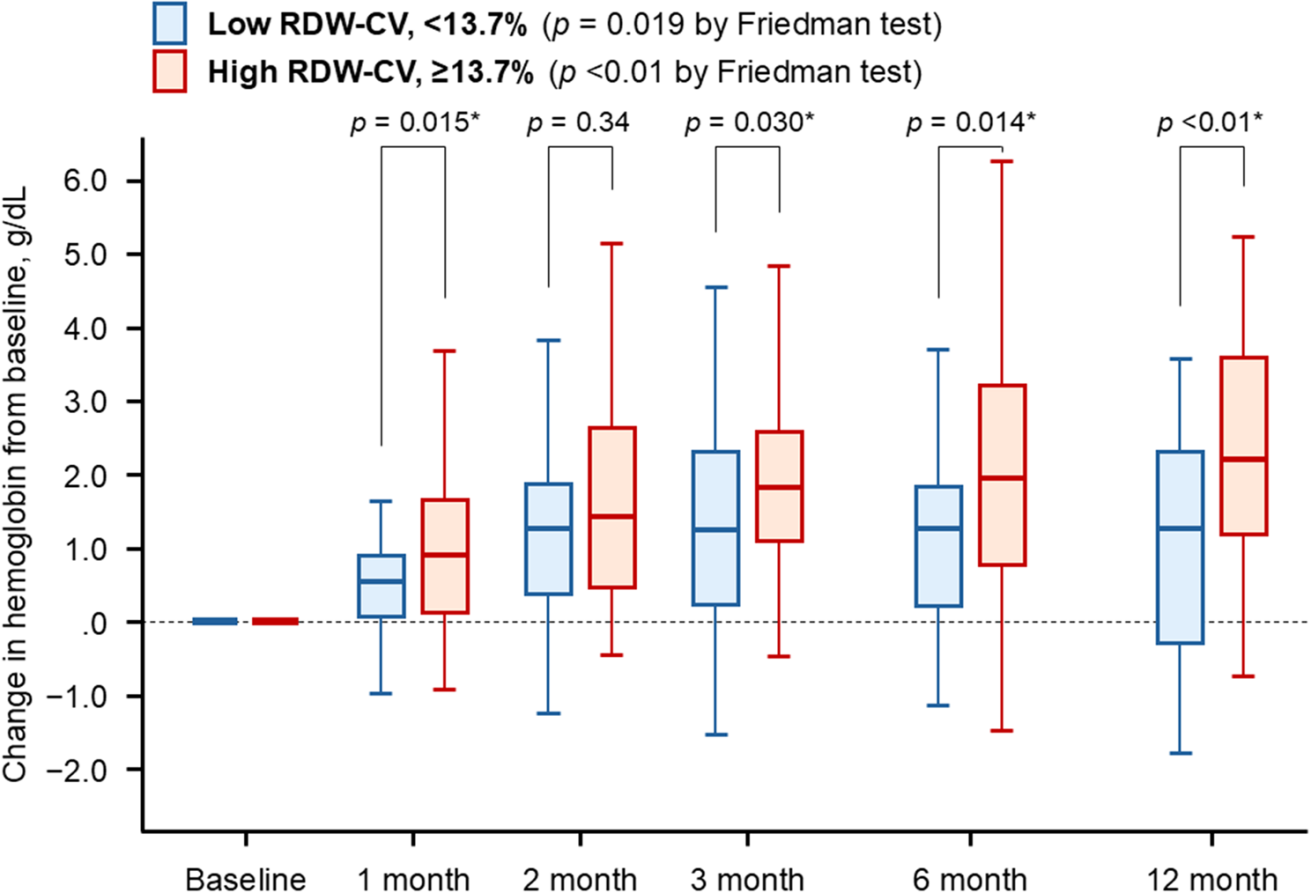
Change in hemoglobin from baseline stratified by the calculated cutoff of RDW-CV. Change in hemoglobin from baseline remained to increase in patients with higher RDW-CV. *p < 0.05 by Mann-Whitney *U* test. Variables were compared between the two groups in each timing. The bars represent the 10th percentile, 25th percentile, median, 75th percentile, and 90th percentile from bottom to top, respectively

Hemoglobin levels significantly improved during the 12-month HIF-PH inhibitor therapy in both RDW-CV groups (lower RDW-CV group: *p* = 0.019, higher RDW-CV group: *p* < 0.01; Fig. 4). However, the magnitude of hemoglobin improvement (change from baseline) was greater among patients with higher RDW-CV. The trajectory of absolute hemoglobin levels stratified by RDW-CV is illustrated in Supplementary Fig. 5. Baseline hemoglobin was lower in the high RDW-CV group, whereas hemoglobin levels increased with no significant difference between two groups during the 12-month therapeutic period.

### Trajectory of RDW-CV

In the whole cohort, RDW-CV trended to increase during the first two months and decreased gradually (Supplementary Fig. 6).

### Impact of baseline RDW-CV on the secondary outcomes

**Fig. 5:**
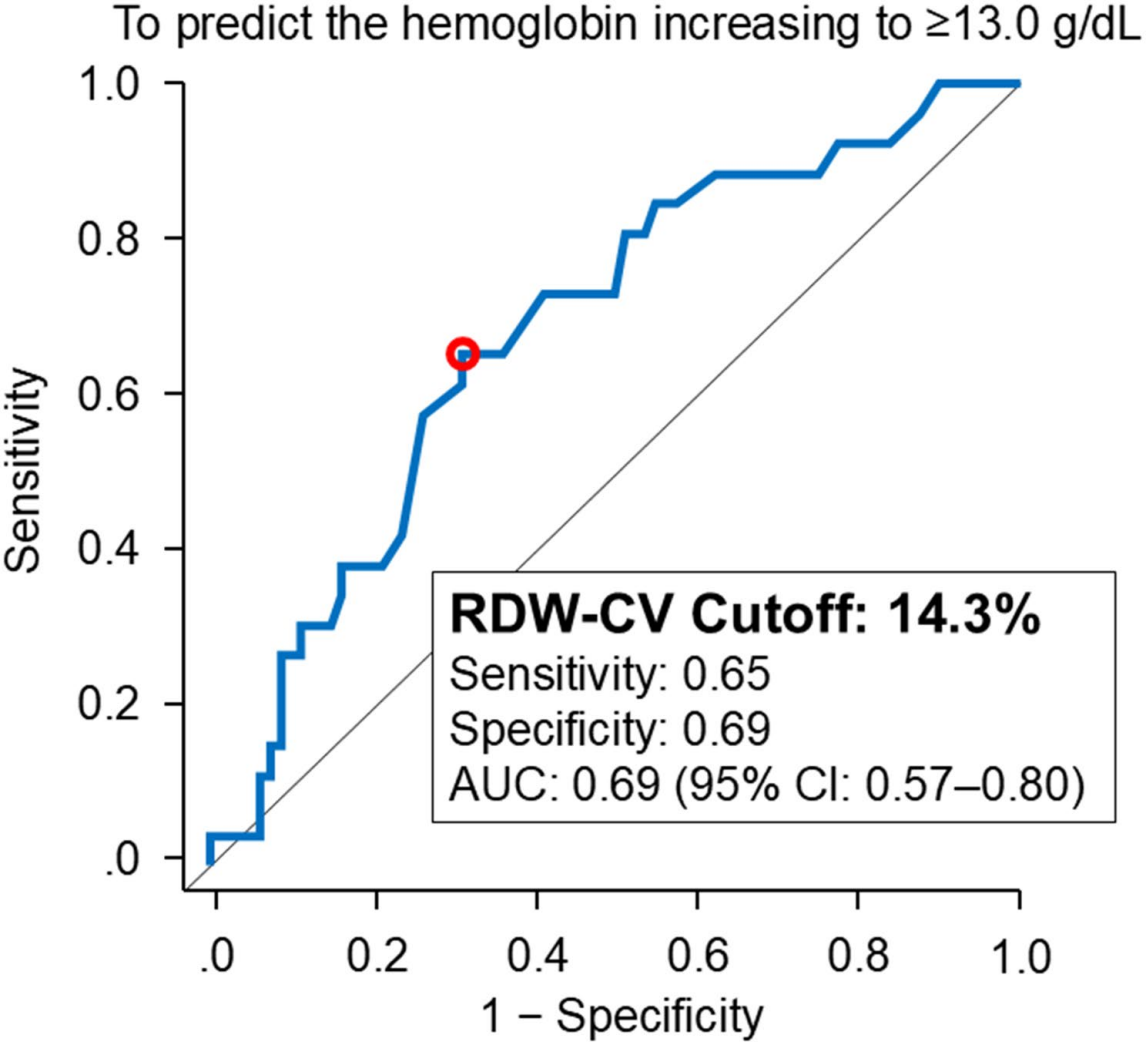
Receiver operating characteristics curves for RDW-CV in relation to secondary outcome. ROC analysis was performed to evaluate the predictive performance of RDW-CV for the secondary outcome: the hemoglobin increasing to 13.0 g/dL or higher. RDW indicates red cell distribution width; CV, coefficient of variation; AUC, area under the curve; CI, confidence interval

**Fig. 6:**
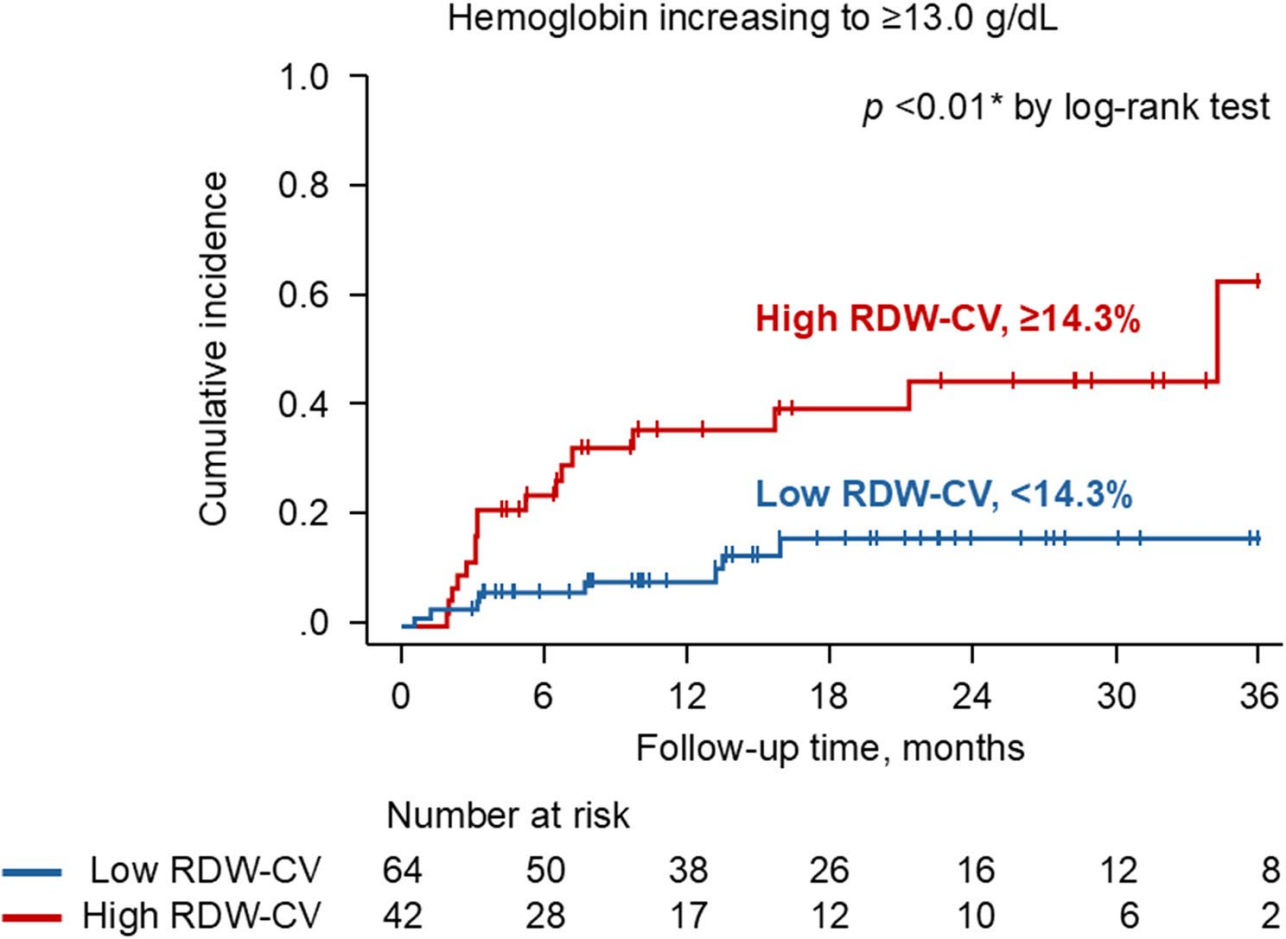
Cumulative incidence of the hemoglobin increasing to 13.0 g/dL or higher stratified by RDW-CV. RDW indicates red cell distribution width; CV, coefficient of variation. The patients were stratified by RDW-CV with a cutoff value of 14.3%. Two curves were compared by log-rank test. *p < 0.05

Patients were followed for a median of 1.6 [0.8–2.4] years after initiation of HIF-PH inhibitors. Twenty-six patients experienced an excessive increase in hemoglobin levels 13.0 g/dL or higher. Baseline RDW-CV independently predicted this outcome, with an adjusted hazard ratio of 1.29 (95% confidence interval 1.04–1.60, *p* = 0.020; Table 3). The RDW-CV cutoff for predicting this secondary outcome (14.3%) was higher than that of the primary outcome (13.7%), with an area under the curve of 0.69 (95% confidence interval 0.57–0.80), sensitivity of 0.65, and specificity of 0.69 (Fig. 5). Higher RDW-CV was significantly associated with a higher cumulative incidence of this secondary outcome (*p* < 0.01; Fig. 6).

**Table 3:** Impact of baseline RDW-CV on predict the hemoglobin increasing to 13.0 g/dL or higher

Explanatory variables	Hazard ratio	95% confidence interval	*p* value
**Univariable analysis**			
Age, years	1.00	0.97–1.03	0.92
Male sex	2.65	1.13–6.24	0.026*
Body mass index, kg/m^2^	0.84	0.74–0.96	< 0.01*
Estimated GFR, mL/min/1.73 m^2^	1.00	0.97–1.04	0.90
Serum albumin, g/dL	0.67	0.38–1.18	0.17
Hemoglobin, g/dL	1.08	0.73–1.61	0.69
Serum ferritin, ng/mL	1.00	0.99–1.00	0.19
Transferrin saturation, %	0.96	0.91–1.01	0.090
RDW-CV, %	1.34	1.12–1.61	< 0.01*
Oral iron supplements during the observation period	2.48	1.04–5.92	0.040*
**Multivariable analysis**			
**Model 1 (Age + Sex)**			
RDW-CV, %	1.40	1.15–1.71	< 0.01*
**Model 2 (Model 1 + Body mass index)**			
RDW-CV, %	1.31	1.06–1.60	0.011*
**Model 3 (Model 2 + Oral iron supplements)**			
RDW-CV, %	1.29	1.04–1.60	0.020*

RDW indicates red cell distribution width; CV, coefficient of variation; GFR, glomerular filtration rate

*p < 0.05 by Cox proportional hazard ratio regression analysis

In total, 29 patients experienced either all-cause mortality (*n* = 16) or progression to end-stage renal disease (*n* = 16). Baseline RDW-CV did not significantly stratify these outcomes (hazard ratio of 0.97, *p* = 0.80; Table 4a). Additionally, 22 patients demonstrated a ≥ 50% decline in estimated GFR, which was also not significantly stratified by baseline RDW-CV (hazard ratio of 1.06, *p* = 0.62; Table 4b). Similarly, the occurrence of ≥ 25% estimated GFR decline showed no significant association with baseline RDW-CV (hazard ratio of 0.95, *p* = 0.45; Table 4c).

**Table 4a:** Impact of baseline RDW-CV on all-cause mortality and/or end-stage renal disease required Dialysis

Explanatory variables	Hazard ratio	95% confidence interval	*p* value
**Univariable analysis**			
Age, years	0.98	0.96–1.00	0.013*
Male sex	1.40	0.67–2.95	0.37
Body mass index, kg/m^2^	1.00	0.93–1.08	0.94
Estimated GFR, mL/min/1.73 m^2^	0.95	0.91–0.99	< 0.01*
Serum albumin, g/dL	0.52	0.31–0.89	0.017*
Hemoglobin, g/dL	0.96	0.69–1.34	0.81
Serum ferritin, ng/mL	1.00	1.00–1.00	0.64
Transferrin saturation, %	1.03	0.99–1.07	0.11
Logarithm of serum CRP, mg/dL	1.27	0.74–2.18	0.39
Logarithm of plasma BNP, pg/mL	1.07	0.39–2.95	0.90
RDW-CV, %	0.97	0.78–1.21	0.80

RDW indicates red cell distribution width; CV, coefficient of variation; GFR, glomerular filtration rate; CRP, C-reactive protein; BNP, B-type natriuretic peptide

*p < 0.05 by Cox proportional hazard ratio regression analysis

**Table 4b:** Impact of baseline RDW-CV on cumulative incidence of ≥ 50% decrease in estimated GFR

Explanatory variables	Hazard ratio	95% confidence interval	*p* value
**Univariable analysis**			
Age, years	0.97	0.95–0.99	< 0.01*
Male sex	1.26	0.54–2.95	0.60
Body mass index, kg/m^2^	1.05	0.98–1.12	0.15
Estimated GFR, mL/min/1.73 m^2^	1.01	0.97–1.05	0.75
Serum albumin, g/dL	0.38	0.20–0.70	< 0.01*
Hemoglobin, g/dL	0.80	0.51–1.24	0.31
Serum ferritin, ng/mL	1.00	1.00–1.00	0.14
Transferrin saturation, %	1.03	0.99–1.07	0.16
Logarithm of serum CRP, mg/dL	1.60	0.88–2.93	0.12
Logarithm of plasma BNP, pg/mL	1.38	0.43–4.43	0.59
RDW-CV, %	1.06	0.85–1.31	0.62

RDW indicates red cell distribution width; CV, coefficient of variation; GFR, glomerular filtration rate; CRP, C-reactive protein; BNP, B-type natriuretic peptide

*p < 0.05 by Cox proportional hazard ratio regression analysis

**Table 4c:** Impact of baseline RDW-CV on cumulative incidence of ≥25% decrease in estimated GFR

Explanatory variables	Hazard ratio	95% confidence interval	pvalue
**Univariable analysis**			
Age, years	0.98	0.97–1.00	0.014*
Male sex	0.92	0.55–1.52	0.73
Body mass index, kg/m^2^	1.03	0.97–1.08	0.36
Estimated GFR, mL/min/1.73 m^2^	1.01	0.99–1.04	0.27
Serum albumin, g/dL	0.71	0.48–1.04	0.075
Hemoglobin, g/dL	0.96	0.75–1.23	0.74
Serum ferritin, ng/mL	1.00	1.00–1.00	0.018*
Transferrin saturation, %	1.02	0.99–1.04	0.27
Logarithm of serum CRP, mg/dL	1.32	0.94–1.85	0.10
Logarithm of plasma BNP, pg/mL	1.37	0.69–2.73	0.37
RDW-CV, %	0.95	0.82–1.09	0.45

RDW indicates red cell distribution width; CV, coefficient of variation; GFR, glomerular filtration rate; CRP, C-reactive protein; BNP, B-type natriuretic peptide

*p <0.05 by Cox proportional hazard ratio regression analysis

### Other clinical outcomes

The trajectory of dosages of major HIF-PH inhibitors (Daprodustat and Vadadustat) is displayed in Supplementary Fig. 7ab. The dosages were not significantly different between the low and high RDW-CV groups during the therapeutic period (Supplementary Table 1).

**Fig. 7:**
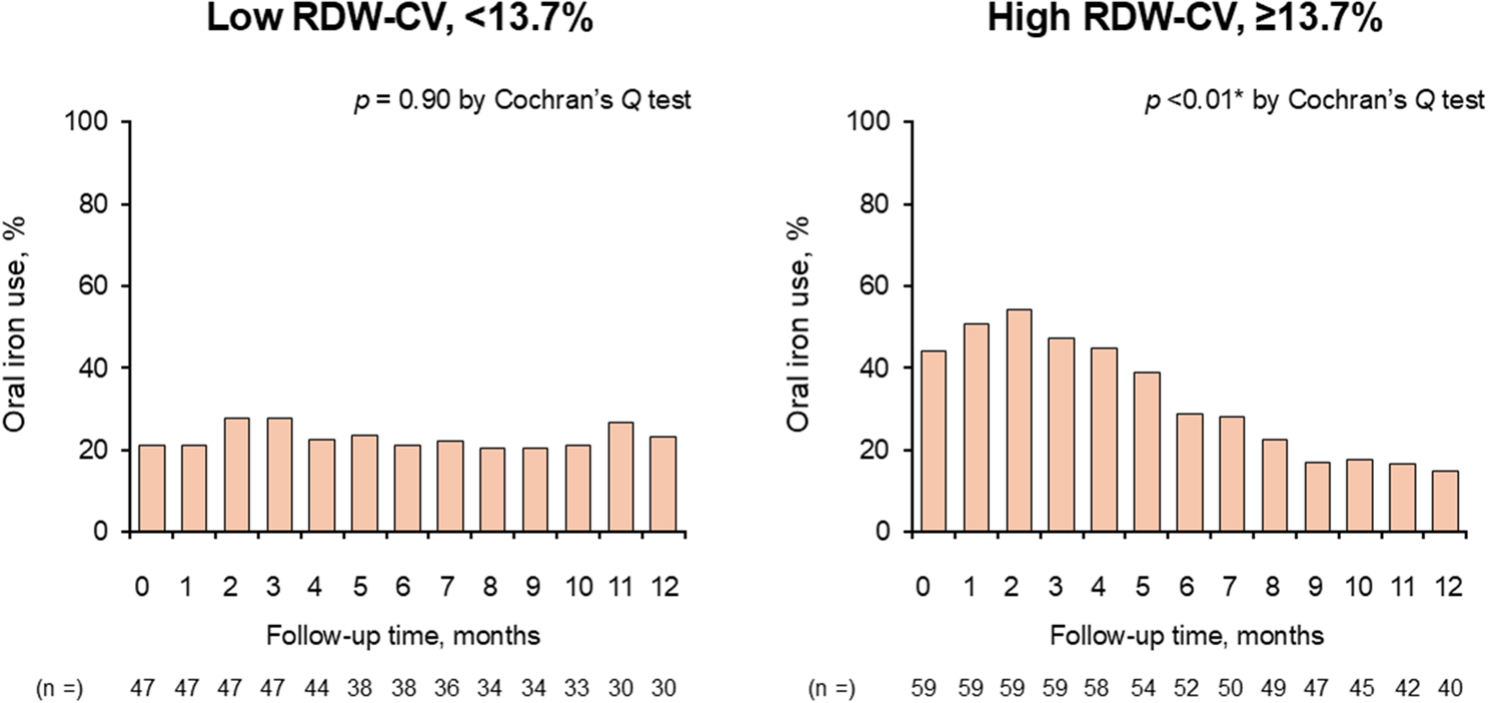
Changes in the percentage of patients receiving oral iron supplements in the two groups. RDW indicates red cell distribution width; CV, coefficient of variation

The prevalence of iron supplementation during 1-year therapeutic period is displayed in Fig. 7. Its prevalence was highest at 2 months (27.7% versus 54.2% in the low and high RDW-CV groups, respectively, *p* < 0.01), whereas they were 23.3% versus 15.0% at 1-year follow-up (*p* = 0.54).

During the therapeutic period, 36 patients encountered cardiovascular diseases (24 heart failures, 3 strokes, 8 arterial diseases, 3 pulmonary hypertension). No patients had thromboembolism, 5 patients had bleeding, 5 patients had hepatic injury, 7 patients had ophthalmic disease, and 11 patients had malignancy. The incidence of treatment-emergent adverse events, including the onset of malignancy and mortality, were higher in the high RDW-CV group (Supplementary Table 2).

## Discussion

In the present study, we examined the influence of baseline RDW-CV on achieving hemoglobin concentrations ≥ 10.0 g/dL within the initial three months following the commencement of HIF-PH inhibitors in patients with anemia in CKD. Elevated baseline RDW-CV independently predicted the primary outcome, with an identified optimal threshold value of 13.7%. Furthermore, an RDW-CV threshold of 14.3% were associated with an increased risk of excessively elevated hemoglobin concentrations of 13.0 g/dL or higher.

### How to define response to HIF-PH inhibitors

Defining the therapeutic response to HIF-PH inhibitors presents a notable challenge. Previous studies have evaluated hemoglobin trajectories over a three-month course of HIF-PH inhibitor therapy [8, 24]. In the present study, the maximum follow-up duration was extended up to four years. However, numerous clinical events during such prolonged observation periods—such as iron deficiency, hemorrhagic episodes, hemodilution due to heart failure, and anemia associated with infections—may confound hemoglobin levels. Therefore, to mitigate the influence of these factors, we defined the increase and maintenance of hemoglobin levels at 10.0 g/dL or higher during the initial three months as the primary outcome.

### Predictors of responses to HIF-PH inhibitor

In real-world clinical settings, a subset of patients exhibits refractoriness to HIF-PH inhibitor therapy. However, there is a paucity of research exploring predictors of therapeutic response, except for a limited small-scale study that identified baseline MCV as a potential predictor [25], and a report suggesting that the value of hepcidin at 1 month after treatment initiation could serve as a predictor of therapeutic response [26]. In the present study, we hypothesized that RDW-CV could serve as a novel predictive marker, owing to its established association with iron metabolism and systemic inflammation. The predictive performance of RDW-CV surpassed MCV (Supplementary Fig. 4).

### Association between RDW-CV and response to HIF-PH inhibitors

HIF-PH inhibitors promote the synthesis of endogenous erythropoietin, regulate iron metabolism, modulate systemic inflammation, and reduce hepcidin levels—thereby enhancing intestinal iron absorption and mobilization of stored iron [14].

Patients with elevated RDW-CV were more likely to have comorbid heart failure and exhibit more advanced states of malnutrition [15, 27]. Additionally, individuals with higher RDW-CV demonstrated lower transferrin saturation, suggesting impaired iron utilization [28]. Chronic heart failure is frequently associated with resistance to ESAs [29] and disrupted iron metabolism [30], both of which contribute to the progression of heart failure and renal dysfunction in the pathophysiological cycle known as the cardio-renal-anemia syndrome [31].

Given that HIF-PH inhibitors target these multifactorial abnormalities—including impaired erythropoiesis, iron dysregulation, and systemic inflammation—patients with elevated RDW-CV may, paradoxically, represent optimal candidates for HIF-PH inhibitors, having sufficient “therapeutic reserve”. In this context, RDW-CV may not simply represent anisocytosis itself, but rather may function as a composite surrogate of several co-existing pathological states, including iron dysmetabolism, inflammation, and the severity of anemia.

### Clinical implication of our findings

A high baseline RDW-CV demonstrated high specificity in predicting improvement of anemia in CKD in response to HIF-PH inhibitor therapy. Patients with elevated RDW-CV often present with comorbid conditions such as heart failure and malnutrition—both of which are commonly associated with resistance to conventional anemia treatments. Despite this, such individuals may be particularly well-suited for HIF-PH inhibitor therapy due to the multifaceted mechanisms of action targeting iron metabolism, inflammation, and erythropoiesis. In patients with elevated baseline RDW-CV, RDW-CV trended to decrease during HIF-PH inhibitor therapy, representing the impact of HIF-PH inhibitor on improving the “nature” of anemia.

Conversely, patients with extremely higher RDW-CV exhibit an increased risk of excessive hemoglobin elevation during treatment, which may predispose them to cardiovascular complications. Therefore, close monitoring of hemoglobin levels is essential. Even when baseline hemoglobin levels are low, initiating treatment with a lower starting dosage of HIF-PH inhibitor and gradual titration is strongly recommended to mitigate potential adverse effects.

### Limitations

Several potential limitations should be acknowledged. The analysis was based on a moderately sized cohort from a single center, which may limit the generalizability of the findings. It is possible that some comparison analyses would have reached statistical significance with a larger sample size. The study employed a retrospective observational design, introducing inherent biases and limiting the ability to establish robust causality.

Approximately one quarter of patients had been previously treated with ESAs before switching to HIF-PH inhibitors, and it is possible that the biological effect of ESA exposure on erythropoiesis and RDW-CV may have persisted for some time during HIF-PH inhibitor therapy. Because the timing and dose intensity of prior ESA use varied among individuals, residual ESA influence could not be completely excluded, and this represents another limitation of our retrospective dataset. Multiple types of HIF-PH inhibitors were used in this study, and potential differences in their pharmacologic effects may have influenced the results [24]. Notably, Roxadustat was excluded from the analysis because it has a considerably longer half-life and is administered every other day, making it difficult to compare directly with other daily-administered agents. The dosage titration of HIF-PH inhibitors was determined at the discretion of the attending physicians, potentially introducing variability in treatment intensity.

We did not follow dynamic trajectory of ferritin, hepcidin, and inflammatory parameters, limiting the robustness of our conclusions. Other unevaluated hemoglobin-related parameters such as reticulocyte hemoglobin content, hypochromic red cell ratio, or hepcidin kinetics may also provide additional information regarding iron availability and erythropoietic activity during HIF-PH inhibitor therapy.

## Conclusions

A higher baseline RDW-CV was independently associated with a favorable therapeutic response to HIF-PH inhibitor treatment in patients with anemia and CKD.

## Supplementary Information

Below is the link to the electronic supplementary material.

Supplementary Material 1

## Data Availability

The datasets used and analysed during the current study are available from the corresponding author on reasonable request.
