# Supplementary material for: Impact of red blood cell distribution width on response to HIF-PH inhibitor in improving anemia and clinical outcomes in patients with chronic kidney disease
Source: BMC Nephrol. 2025 Dec 6;27:33. doi: 10.1186/s12882-025-04675-2 (PMC12797824; doi:10.1186/s12882-025-04675-2)
Supplement: Supplementary file 1 — Supplementary Material 1 [file 12882_2025_4675_MOESM1_ESM.pdf]

Article title:
Impact of red blood cell distribution width on response to HIF-PH inhibitor in improving anemia and clinical outcomes in patients with chronic kidney disease

Journal name:
BMC Nephrology

Author names:
Sayaka Murai, Kota Kakeshita, Teruhiko Imamura, Tsutomu Koike, Hidenori Yamazaki, and Koichiro Kinugawa

Affiliation and e-mail address of the corresponding author:
Teruhiko Imamura, MD PhD FAHA FACC FESC FJCS FHFSA FAPSC FACP FICA FASA FJCC
The Second Department of Internal Medicine, University of Toyama, Toyama, Japan
teimamu@med.u-toyama.ac.jp

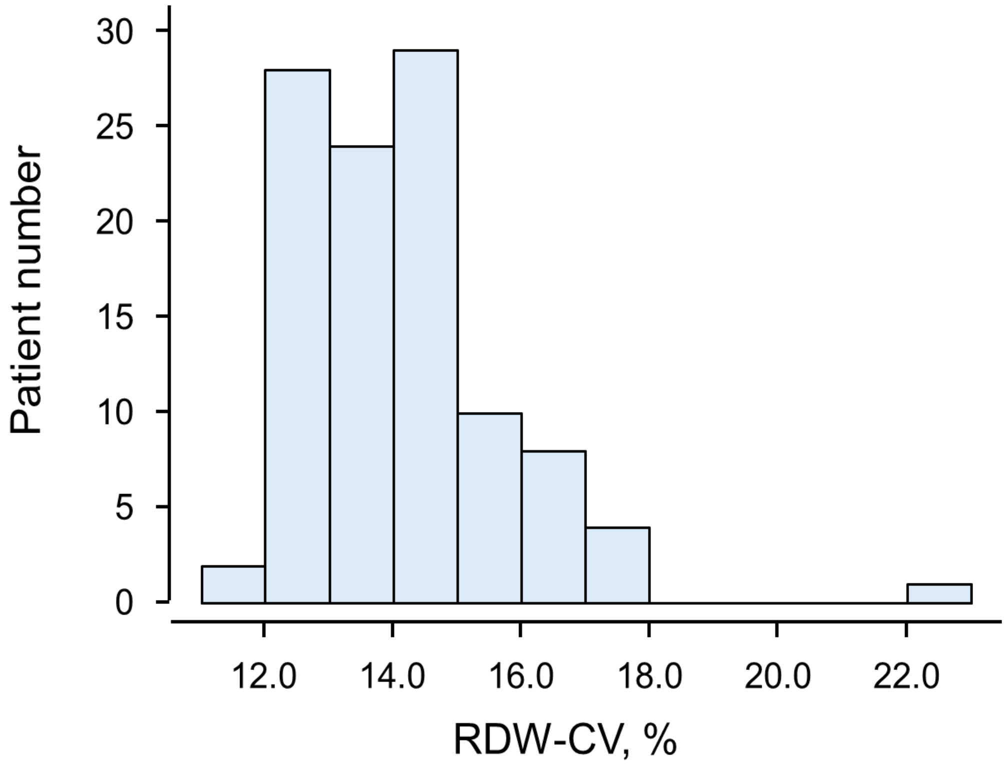

Supplementary Fig. 1 Distribution of RDW-CV
RDW indicates red cell distribution width; CV, coefficient of variation.

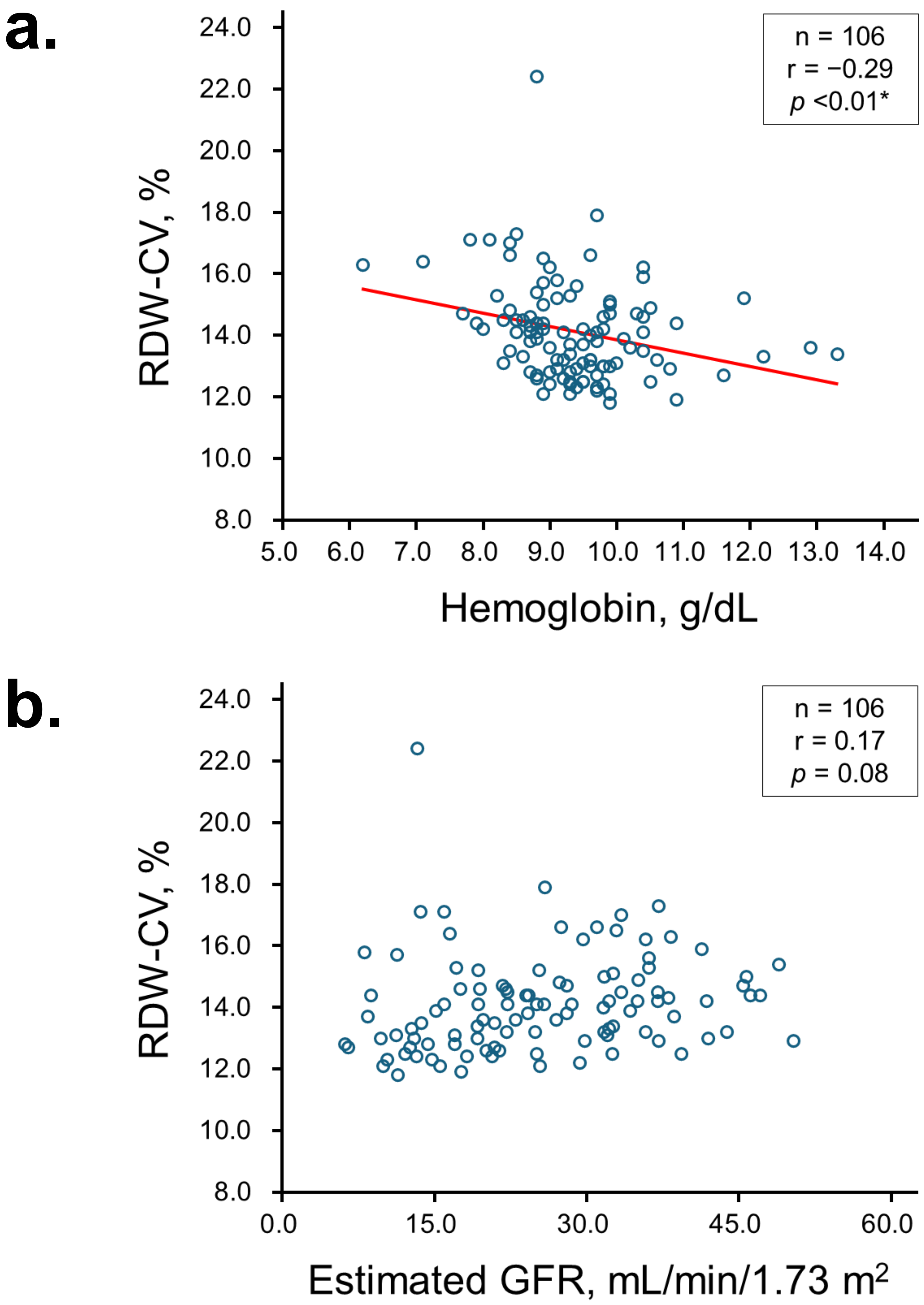

Supplementary Fig. 2 Correlation between hemoglobin and RDW-CV (a), and between estimated GFR and RDW-CV (b)

RDW indicates red cell distribution width; CV, coefficient of variation; GFR, glomerular filtration rate. *p <0.05 by Pearson's correlation coefficient.

Without pre-ESAs

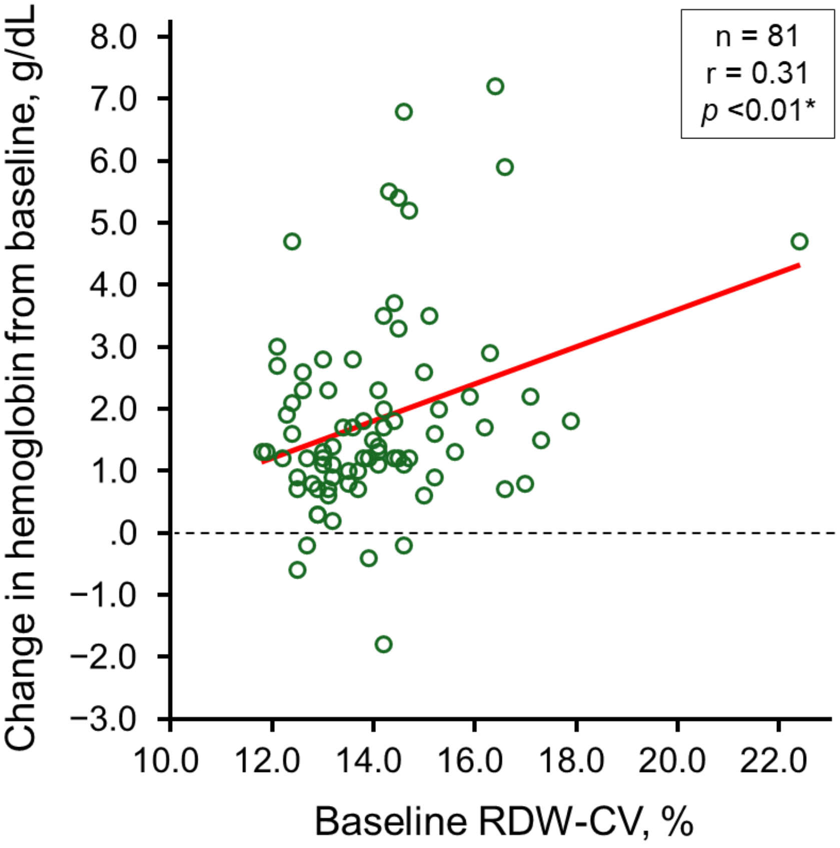

With pre-ESAs

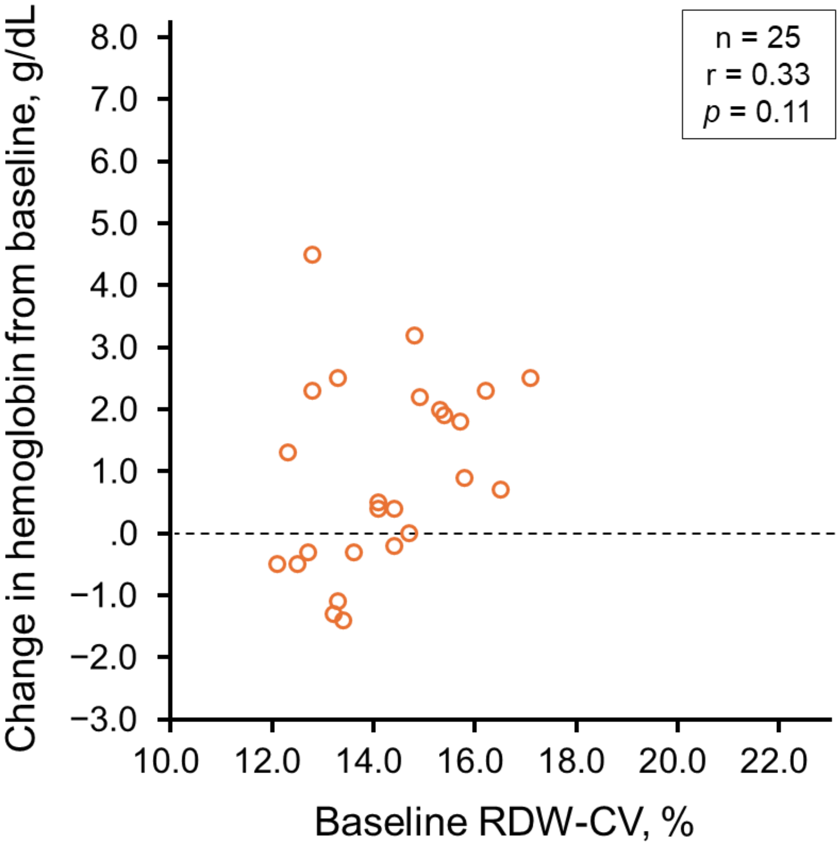

Supplementary Fig. 3 Correlation between baseline RDW-CV and change in hemoglobin during first 3 months by pre-ESAs status

RDW indicates red cell distribution width; CV, coefficient of variation; ESA, erythropoiesis stimulating agent. **p* <0.05 by Pearson's correlation coefficient.

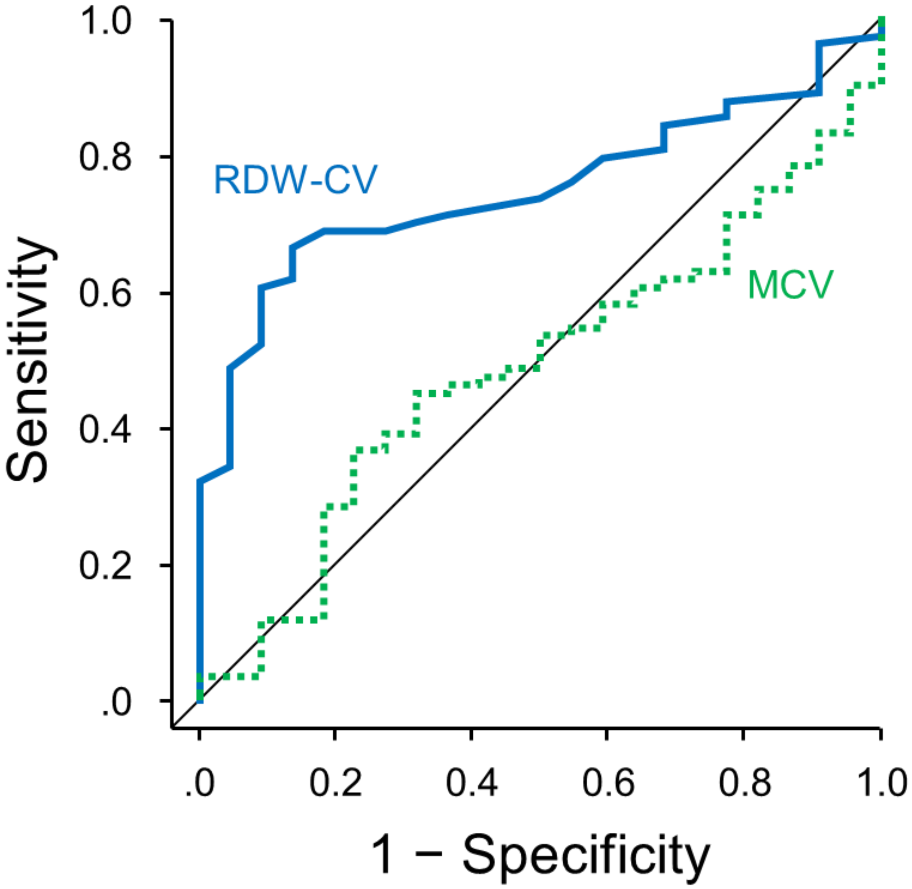

Predictor	AUC	95% CI	*p* value
RDW-CV	0.75	0.66–0.85	reference
MCV	0.49	0.36–0.62	<0.01*

Supplementary Fig. 4 Receiver operating characteristics curves comparing predictive performance of RDW-CV and MCV

The ROC curves compare the predictive performance of RDW-CV (blue solid line) and MCV (green dotted line) for the primary outcome.

RDW indicates red cell distribution width; CV, coefficient of variation; MCV, mean corpuscular volume; AUC, area under the curve; CI, confidence interval. *p <0.05 by DeLong's test.

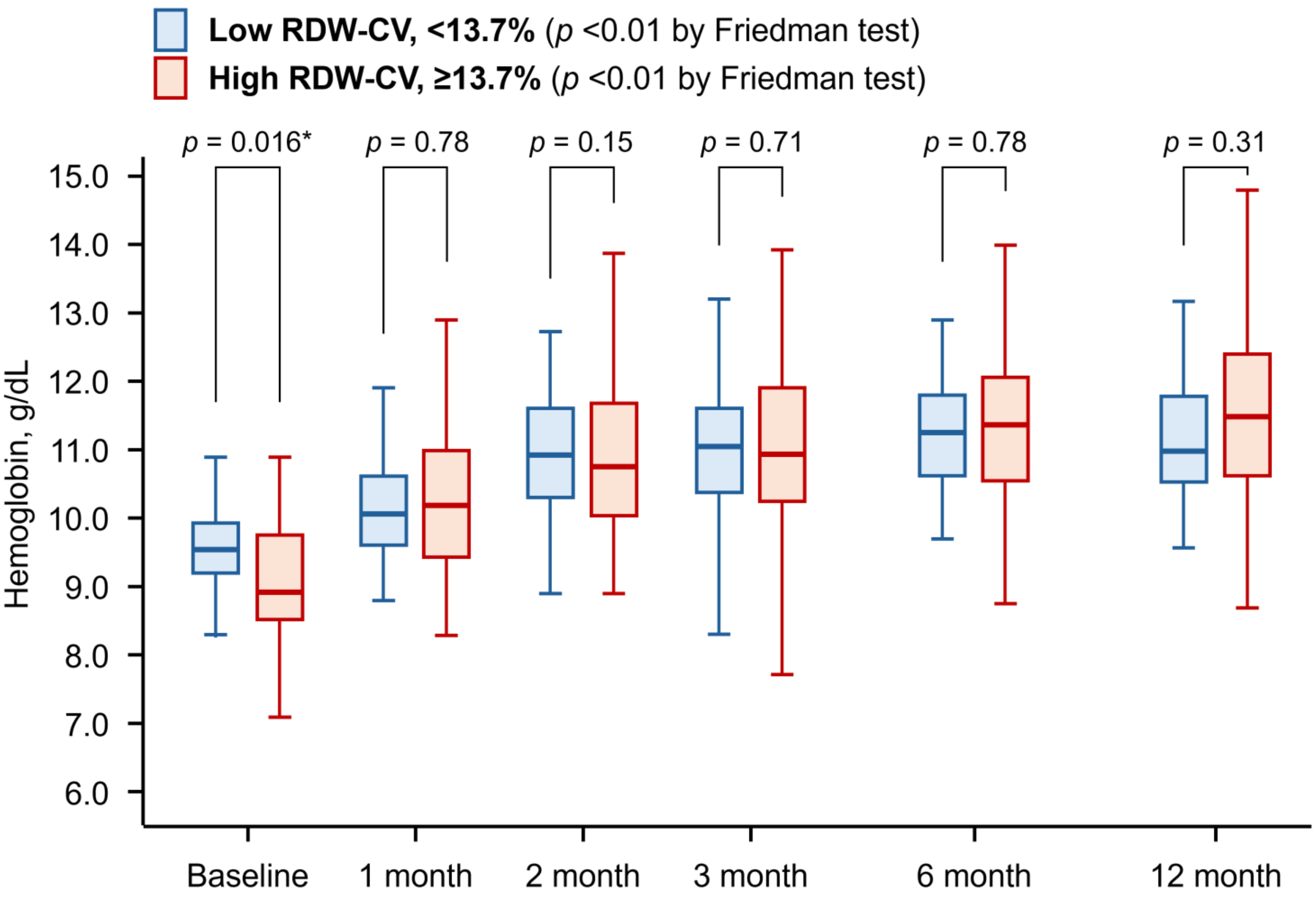

Supplementary Fig. 5 Change in hemoglobin stratified by the cutoff of RDW-CV

Baseline hemoglobin was significantly lower and change in hemoglobin from baseline remained to increase in patients with higher RDW-CV. *$p < 0.05$ by Mann-Whitney *U* test. Variables were compared between the two groups in each timing. The bars represent the 10th percentile, 25th percentile, median, 75th percentile, and 90th percentile from bottom to top, respectively.

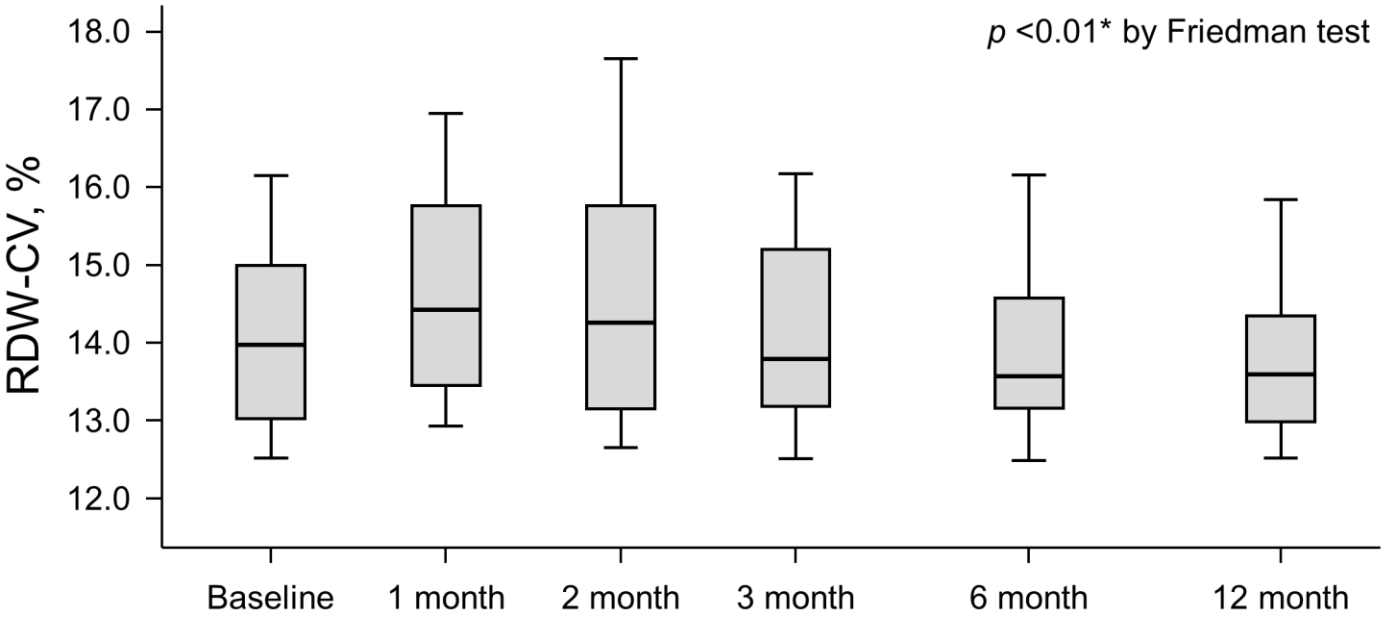

Supplementary Fig. 6 Change in RDW-CV in total patients
RDW indicates red cell distribution width; CV, coefficient of variation. The bars represent the 10th percentile, 25th percentile, median, 75th percentile, and 90th percentile from bottom to top, respectively. *$p < 0.05$ by Friedman test.

a. **Daprodustat**

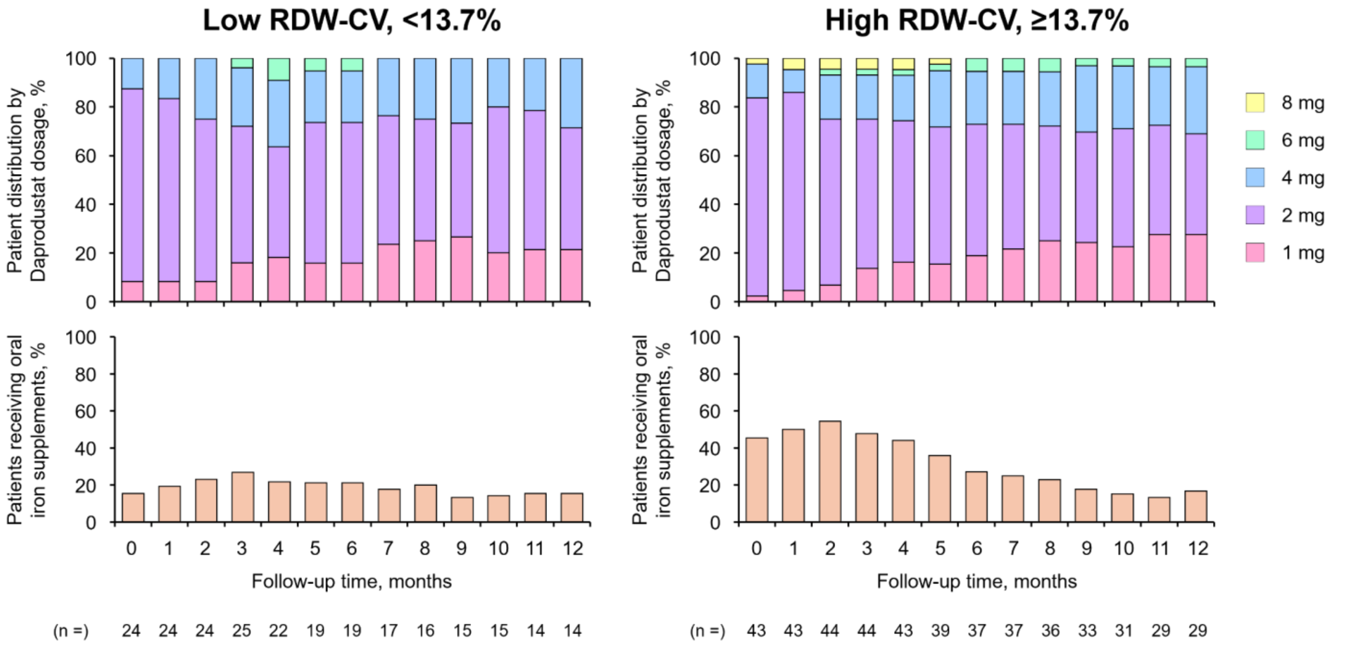

b. **Vadadustat**

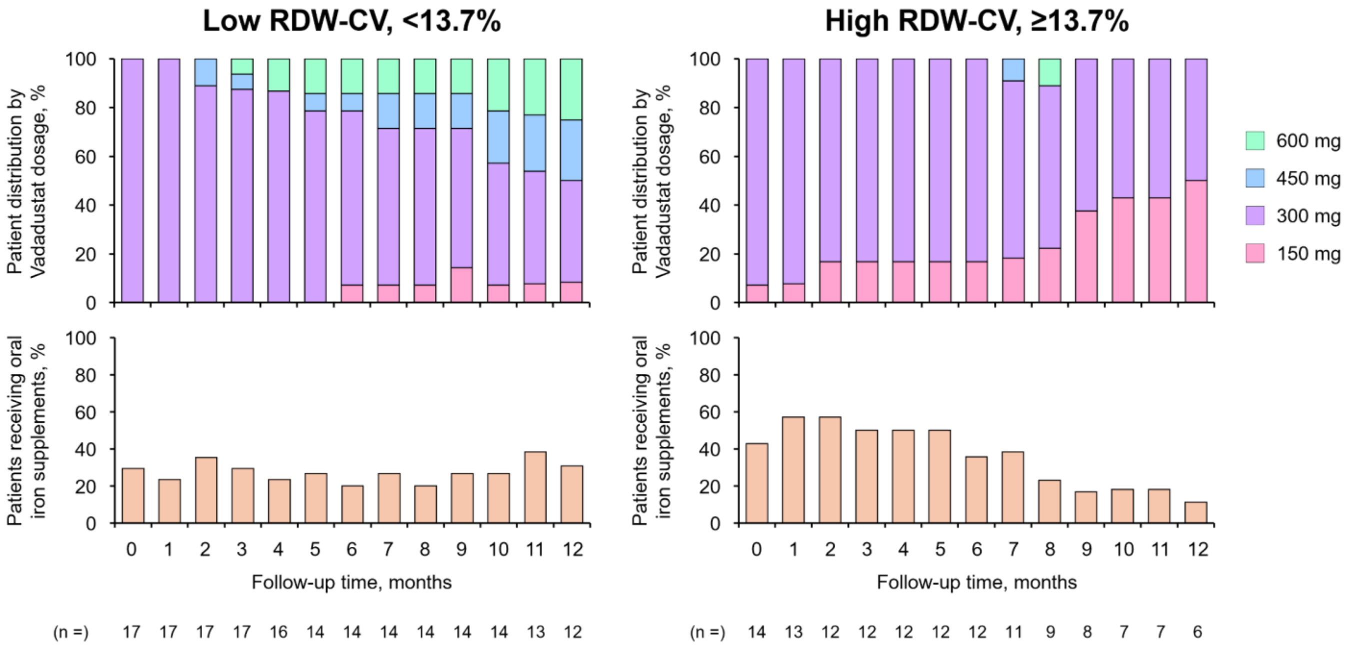

Supplementary Fig. 7 Patient distribution by HIF-PH inhibitor dosage and oral iron supplement use

Changes in patient distribution are shown according to HIF-PH inhibitor dosage and oral iron supplement use. Daprodustat (a) and Vadadustat (b).

RDW indicates red cell distribution width; CV, coefficient of variation.

Supplementary Table 1 Changes in HIF-PH inhibitor dosage

	Total	Low RDW-CV (<13.7%)	High RDW-CV (≥13.7%)	Number of patients (Low group/High group)	*p* value
**Daprodustat dosage, mg/day**					
Day 0	2 [2–2]	2 [2–2]	2 [2–2]	24/43	0.38
Month 3	2 [2–4]	2 [2–4]	2 [2–3]	25/44	0.99
Month 6	2 [2–4]	2 [2–3]	2 [2–4]	19/37	0.92
Month 12	2 [2–4]	2 [2–4]	2 [1–4]	14/29	0.95
**Vadadustat dosage, mg/day**					
Day 0	300 [300–300]	300 [300–300]	300 [300–300]	17/14	0.30
Month 3	300 [300–300]	300 [300–300]	300 [300–300]	17/12	0.050
Month 6	300 [300–300]	300 [300–300]	300 [300–300]	14/12	0.12
Month 12	300 [300–450]	375 [300–488]	300 [188–300]	12/6	0.083

HIF-PH indicates hypoxia inducible factor-prolyl hydroxylase; RDW, red cell distribution width; CV, coefficient of variation.

Each dosage is presented as median [25th percentile–75th percentile] and compared between the two groups using Mann-Whitney *U* test.

Supplementary Table 2 Treatment-emergent adverse events

	**Total (N = 106)**	**Low RDW-CV (<13.7%) (n = 47)**	**High RDW-CV (≥13.7%) (n = 59)**	***p* value**
Cardiovascular diseases	36 (34%)	14 (30%)	22 (37%)	0.54
Acute coronary syndrome	0	0	0	1.0
Hospitalization for heart failure	24 (23%)	10 (21%)	14 (24%)	0.82
Stroke	3 (3%)	2 (4%)	1 (2%)	0.58
Arterial disease	8 (8%)	2 (4%)	6 (10%)	0.30
Pulmonary hypertension	3 (3%)	0	3 (5%)	0.25
Thrombosis	0	0	0	1.0
Bleeding	5 (5%)	1 (2%)	4 (7%)	0.38
Gastrointestinal hemorrhage	3 (3%)	0	3 (5%)	0.25
Ophthalmological disease	7 (7%)	2 (4%)	5 (8%)	0.46
Retinal hemorrhage	1 (1%)	0	1 (2%)	1.0
Diabetic retinal edema	3 (3%)	1 (2%)	2 (3%)	1.0
Glaucoma	1 (1%)	0	1 (2%)	1.0
Macular degeneration	2 (2%)	1 (2%)	1 (2%)	1.0
Malignant or unspecified tumors	11 (10%)	1 (2%)	10 (17%)	0.021*
Gastric or colorectal cancer	4 (4%)	0	4 (7%)	0.13
Lung cancer	2 (2%)	1 (2%)	1 (2%)	1.0
Prostate or bladder cancer	2 (2%)	0	2 (3%)	0.50
Hepatic function disorder	5 (5%)	3 (6%)	2 (3%)	0.65
Itchiness	1 (1%)	1 (2%)	0	0.44
End-stage renal disease that required dialysis	16 (15%)	9 (19%)	7 (12%)	0.41
Any treatment-emergent adverse event resulting in death	16 (15%)	2 (4%)	14 (24%)	<0.01*

RDW indicates red cell distribution width; CV, coefficient of variation. Variables are presented as numbers (percentage) and compared between the two groups using Fisher’s exact test.
